# Oral Diseases and Brain Pathologies: A Systematic Review with Narrative Synthesis of Clinical, Neuroimaging, and Mechanistic Evidence

**DOI:** 10.3390/biomedicines14040768

**Published:** 2026-03-28

**Authors:** Marines Vega Sanchez, Francisco Córdova, Maria Rodríguez Tatés, Luis Chauca Bajaña, Diego Quiguango Farías, María Flores Araque, Byron Velásquez Ron

**Affiliations:** 1 Carrera de Odontología, Facultad de Odontología, Universidad de Las Américas Ecuador (UDLA), Colon y 6 de Diciembre, Quito 170516, Ecuador; marines.vega@udla.edu.ec (M.V.S.); francisco.cordova@udla.edu.ec (F.C.); gaby.rodriguezt1@gmail.com (M.R.T.); maria.flores.araque@udla.edu.ec (M.F.A.); 2 Dental Sciences, College Dentistry, University of Guayaquil, Guayaquil 090514, Ecuador; luischauk@hotmail.com; 3 Carrera Ciencias de la Salud, Universidad de Las Américas (UDLA), Quito 170516, Ecuador; diego.quiguango@udla.edu.ec; 4 Department Prosthesis Research, Av. Colón y 6 Diciembre, Quito 170516, Ecuador

**Keywords:** oral health, periodontitis, brain abscess, glioma, oral–brain axis, inflammation, extracellular vesicles

## Abstract

**Background:** Oral diseases such as periodontitis, dental infections, and oral dysbiosis have been increasingly associated with systemic conditions. Emerging evidence suggests a potential relationship between oral health and neurological disorders, including brain abscesses, structural brain alterations, and gliomas. However, the strength and mechanisms of these associations remain incompletely understood. **Objective:** To systematically review clinical, neuroimaging, genetic, and mechanistic evidence linking oral diseases with brain pathologies. **Methods:** A systematic literature search was conducted in PubMed, Scopus, Web of Science, and EBSCO, with complementary screening of SciELO, Redalyc, and LILACS databases. Studies evaluating associations between oral diseases (periodontitis, dental infections, caries, or oral microbiota alterations) and neurological outcomes were considered. Eligible study designs included observational clinical studies, Mendelian randomization analyses, neuroimaging studies, and experimental investigations. Seventeen studies met the inclusion criteria. Due to the substantial heterogeneity in study designs, outcomes, and effect metrics, quantitative meta-analysis was not feasible. Findings were therefore synthesized using a structured narrative approach following PRISMA guidelines. **Results:** Clinical studies consistently identified odontogenic infections as a relevant source of brain abscesses, frequently originating from chronic or clinically silent dental foci. Neuroimaging and genetic studies reported associations between poor oral health indicators and structural brain alterations, including reduced cortical thickness and white matter abnormalities. Experimental investigations suggested potential biological mechanisms involving microbial dissemination, systemic inflammation, and immune modulation. Virulence factors from *Porphyromonas gingivalis* have been shown to induce inflammatory signaling pathways and immune checkpoint activation in glioma cells. **Conclusions:** The current evidence suggests a possible association between oral diseases and several brain pathologies. Although causality cannot be established, the findings highlight the importance of oral health as a potentially modifiable factor relevant to neurological health. Further longitudinal and mechanistic studies are required to clarify these relationships.

## 1. Introduction

Oral diseases, such as periodontitis, dental caries, and odontogenic infections, represent a significant global public health burden [1,2]. These conditions not only affect the oral cavity but have also been associated with multiple systemic diseases, including cardiovascular disorders, diabetes, cancer, and neurodegenerative diseases [3,4]. In recent years, increasing interest has emerged in understanding the possible relationship between oral health and central nervous system diseases, particularly brain tumors and intracranial inflammatory processes [5]. Brain tumors, such as gliomas, are among the most aggressive neoplasms of the central nervous system, with high morbidity and mortality rates [6]. Despite advances in neurosurgery, radiotherapy, and molecular therapies, prognosis remains poor. In parallel, brain abscesses continue to represent a severe infectious complication, frequently associated with bacteria of oral origin [7]. These findings have prompted the exploration of the oral cavity as a potential reservoir of pathogens and inflammatory mediators affecting the brain [8,9]. Several observational, experimental, and genetic studies suggest that oral dysbiosis, periodontitis, and dental infections may influence neuroinflammation, tumor progression, and structural brain changes [10]. Proposed mechanisms include hematogenous dissemination of oral bacteria, activation of systemic immune responses, release of bacterial lipopolysaccharides, and modulation of inflammatory pathways such as Akt, NF-κB, and pro-inflammatory cytokines [11,12]. Moreover, Mendelian randomization studies have provided genetic evidence supporting a potential causal relationship between oral diseases and cerebral alterations [13], and the available literature is heterogeneous in terms of methodological designs, types of oral exposure, neurological outcomes, and effect metrics, which hinders robust quantitative meta-analyses [14]. Therefore, synthesizing the existing evidence through a narrative systematic review is essential to integrate clinical, microbiological, genetic, and mechanistic findings [15]. The objective of this systematic review is to analyze the association between oral diseases and brain pathologies, including tumors and abscesses, describe the proposed biological mechanisms, and evaluate the quality of the available evidence. This synthesis aims to contribute to a better understanding of the oral–brain axis and its clinical and preventive implications.

## 2. Methods

### 2.1. Study Design and Reporting Guidelines

This systematic review was conducted and reported in accordance with the Preferred Reporting Items for Systematic Reviews and Meta-Analyses (PRISMA 2020) guidelines. The review protocol was prospectively registered in the International Prospective Register of Systematic Reviews (PROSPERO) under registration number CRD420261290918. The completed PRISMA checklist is provided as Supplementary Material [16].

The objective of this review was to synthesize the available evidence regarding the association between oral diseases and brain pathologies, including infectious, structural, and neoplastic outcomes.

### 2.2. Review Question and Eligibility Framework

The review question was structured using the PICO framework:

Population: individuals with oral diseases. Exposure: periodontitis, dental caries, odontogenic infections, or alterations of the oral microbiota. Comparator: individuals without oral disease or with different levels of oral health status. Outcomes: brain abscesses, structural brain alterations (e.g., cortical thickness, white matter abnormalities), or brain tumors (e.g., glioma).

Eligible studies included:

- Observational clinical studies (cohort, case–control, cross-sectional)
- Mendelian randomization analyses
- Neuroimaging studies evaluating brain structural changes
- Experimental or mechanistic studies examining biological pathways linking oral disease with brain pathology

Narrative or systematic reviews were not included as primary analytical studies but were considered when they provided relevant contextual or mechanistic interpretation of the oral–brain relationship.

Studies were excluded if they:

- Lacked a comparator group
- Did not evaluate brain outcomes
- Were purely descriptive opinion papers
- Consisted exclusively of single case reports without microbiological or mechanistic relevance

Only studies published in English or Spanish were considered.

### 2.3. Search Strategy

A systematic literature search was conducted across the following electronic databases:

- PubMed/MEDLINE
- Scopus
- Web of Science
- EBSCO

To broaden coverage of regional literature, additional screening was performed in SciELO, Redalyc, and LILACS.

The search strategy combined terms related to oral diseases and neurological outcomes using Boolean operators. Keywords included combinations of the following:

(“oral disease” OR “oral health” OR “periodontal disease” OR periodontitis OR “dental infection” OR “odontogenic infection” OR “oral microbiota” OR dysbiosis OR “dental caries”) AND (“brain disease” OR “brain disorder” OR “central nervous system” OR glioma OR “brain abscess” OR neuroinflammation OR “white matter hyperintensity” OR “brain imaging” OR MRI).

Reference lists of relevant articles were also screened to identify additional eligible studies.

All retrieved records were screened in two stages. First, titles and abstracts were reviewed to identify potentially relevant studies. Second, full-text articles were assessed for eligibility according to the predefined inclusion criteria.

Reasons for exclusion at the full-text stage included narrative reviews without primary data, letters or editorials, and studies not directly examining brain outcomes.

**Figure 1:**
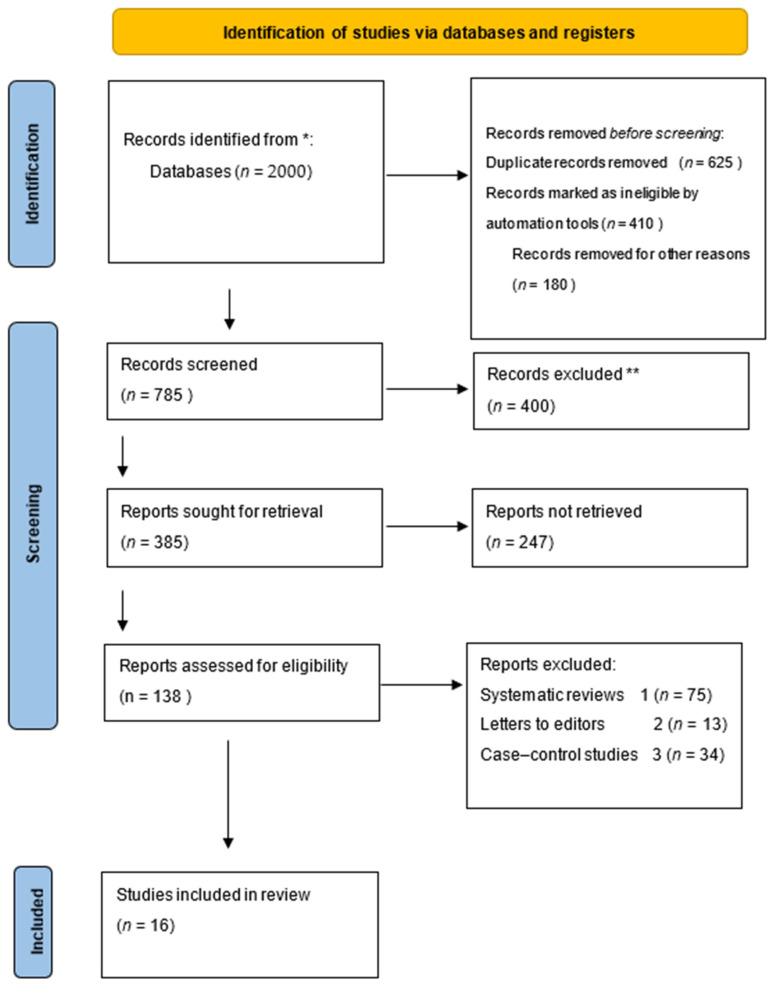
Prisma flow diagram. * Consider, if feasible to do so, reporting the number of records identified from each database or register searched (rather than the total number across all databases/registers). ** If automation tools were used, indicate how many records were excluded by a human and how many were excluded by automation tools.

The study selection process is summarized in the PRISMA flow diagram (Figure 1).

### 2.4. Data Extraction

Data extraction was performed using a standardized data collection form. Extracted variables included:

▪ Study design;
▪ Study population and sample size;
▪ Type of oral exposure or phenotype;
▪ Neurological outcome investigated [17];
▪ Key findings and direction of association;
▪ Study setting and country [18].

These data were used to construct summary tables describing the characteristics and main findings of the included studies.

### 2.5. Evidence Domain Framework and Data Synthesis

Because the included studies differed substantially in design, outcome definitions, and effect metrics, quantitative meta-analysis was not considered appropriate. Instead, a structured narrative synthesis was performed [19].

To facilitate interpretation of heterogeneous evidence, studies were organized into three predefined domains:

1. Clinical infectious evidence linking odontogenic infections with brain abscesses;
2. Neuroimaging and genetic evidence evaluating associations between oral health and structural brain alterations;
3. Experimental and mechanistic studies investigating microbial and immunological pathways [20].

**Figure 2:**
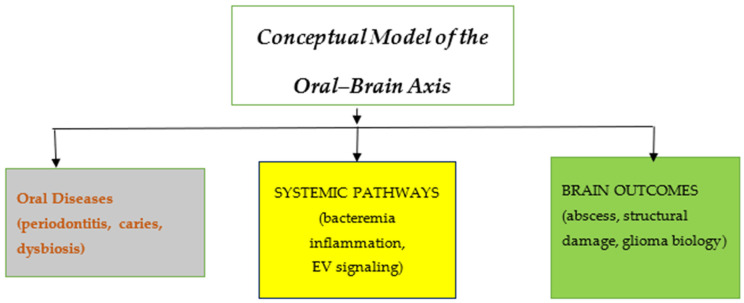
Conceptual model of the oral–brain axis.

Within each domain, findings were summarized according to direction of association, biological plausibility, and methodological characteristics. These routes are illustrated in Figure 2.

Proposed pathways linking oral diseases with neurological outcomes: Periodontal disease, odontogenic infections, and oral microbiota dysbiosis may contribute to systemic dissemination of bacteria, inflammatory signaling, and extracellular vesicle–mediated communication. These mechanisms may influence brain pathologies such as cerebral abscess formation, structural brain alterations, and tumor microenvironment modulation.

Studies were categorized into evidence domains (clinical infectious, neuroimaging/genetic, microbiome, and mechanistic) to facilitate structured narrative synthesis.

## 3. Results

### 3.1. Study Selection

The database search identified 2000 records across the selected electronic databases. After removal of duplicate records and initial automated filtering, 785 records remained for title and abstract screening. Following this stage, 400 records were excluded because they did not meet the eligibility criteria. A total of 385 full-text articles were sought for retrieval, of which 247 were not available or did not meet inclusion criteria. After full-text assessment, 138 studies were evaluated for eligibility. Among these, studies were excluded for the following reasons:

- Narrative reviews without original data (*n* = 75)
- Letters or editorials (*n* = 13)
- Ineligible study designs or lack of appropriate comparator groups (*n* = 34)

Finally, 16 studies met the inclusion criteria and were included in the qualitative synthesis.

### 3.2. Characteristics of Included Studies

The 16 included studies represented diverse methodological designs investigating the relationship between oral diseases and neurological outcomes.

The evidence included:

- Population-based cohort studies
- Case–control studies
- Mendelian randomization analyses
- Neuroimaging investigations
- Experimental mechanistic studies

The main oral exposures investigated were:

- Periodontal disease and periodontal parameters
- Dental caries burden
- Odontogenic infections
- Oral microbiota composition
- Virulence factors from oral pathogens

Neurological outcomes examined across studies included:

- Brain abscess formation
- Structural brain alterations, such as cortical thinning and white matter abnormalities
- Glioma presence or progression

The characteristics of the included studies are summarized in Table 1, which classifies the evidence according to study design, exposure type, neurological outcomes, and evidence domain.

### 3.3. Domain 1: Clinical Evidence Linking Oral Infections with Brain Abscesses

Seven studies evaluated the association between odontogenic infections and brain abscess formation. Population-based cohort studies and retrospective clinical analyses consistently identified oral infections as a relevant source of brain abscesses, particularly in cases where no other infectious focus was identified. In a nationwide Danish cohort, odontogenic foci were detected in approximately one-quarter of brain abscess cases, and oral bacteria belonging to the Streptococcus anginous group were frequently isolated from abscess material. Similarly, retrospective analyses reported that a substantial proportion of cryptogenic brain abscesses were associated with chronic or clinically silent dental infections, including untreated periodontitis and dental caries. Case series and microbiological investigations further demonstrated that typical oral pathogens, including *Fusobacterium nucleatum*, *Parvimonas micra*, and *Porphyromonas gingivalis*, could be isolated from abscess material even when patients did not present overt dental symptoms. These findings suggest that occult odontogenic infections may represent an underrecognized source of cerebral abscess formation (Table 2).

### 3.4. Domain 2: Neuroimaging and Genetic Evidence of Structural Brain Alterations

Three studies investigated associations between oral health indicators and structural brain alterations using neuroimaging or genetic approaches. A Mendelian randomization study demonstrated that genetically predicted dental caries burden was associated with reduced cortical thickness in specific brain regions. These findings suggest a potential relationship between oral health and structural brain integrity. In a large neuroimaging cohort derived from the UK Biobank, poor oral health indicators, such as dentures or loose teeth, were associated with increased white matter hyperintensities and impaired white matter microstructural integrity. These neuroimaging markers are commonly interpreted as indicators of cerebral small vessel disease and chronic inflammatory injury. Although causal inference remains limited, these findings provide preliminary evidence linking oral health status with subclinical brain structural changes (Table 3).

Several studies investigated biological mechanisms that could explain potential links between oral diseases and neurological pathology. Experimental investigations reported that virulence factors derived from *Porphyromonas gingivalis*, particularly gingipains, can induce inflammatory signaling in glioma cells. Laboratory models demonstrated increased IL-6 secretion and upregulation of PD-L1 expression, suggesting potential effects on tumor immune evasion and progression. Additional studies highlighted the role of extracellular vesicles (EVs) released by periodontal tissues and oral pathogens. These vesicles contain inflammatory mediators, microRNAs, and signaling molecules capable of modulating immune responses and angiogenesis. Experimental evidence suggested that EV-derived microRNAs may activate signaling pathways involved in vascular remodeling and inflammatory responses, providing a potential systemic communication mechanism between oral inflammation and distant organs. Together, these findings support the biological plausibility of the proposed oral–brain axis, although direct causal relationships remain to be established (Table 4).

Two Mendelian randomization studies and one large neuroimaging cohort provided evidence linking oral health to structural brain changes. Wang et al. demonstrated that genetically predicted dental caries (DMFS index) was associated with reduced cortical thickness in the superior temporal sulcus, suggesting a potential causal effect of caries on brain morphology [21]. Similarly, Rivier et al. reported that poor oral health proxies (dentures or loose teeth) were associated with increased white matter hyperintensities and impaired white matter integrity. Mendelian randomization analyses supported a possible causal relationship [22].

These findings indicate that oral diseases may contribute to subclinical brain injury, potentially through chronic systemic inflammation or vascular mechanisms

### 3.5. Oral Microbiota and Glioma

**Figure 3:**
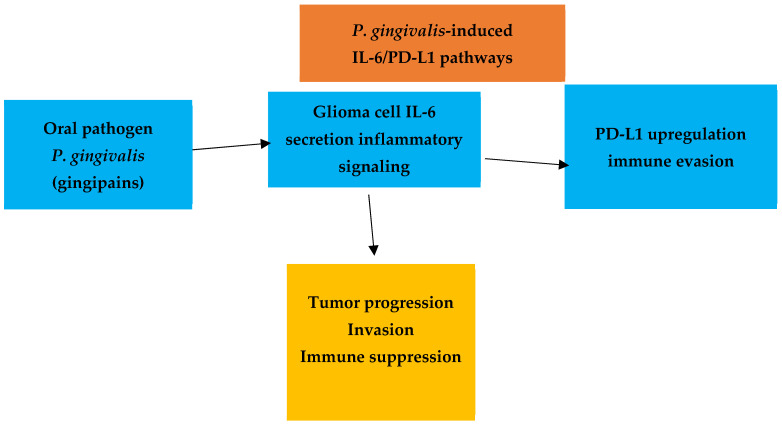
Proposed mechanism linking *Porphyromonas gingivalis* infection to glioma progression. Gingipain antigens may induce IL-6 secretion in glioma cells, leading to PD-L1 upregulation and immune evasion, thereby promoting tumor progression.

Two clinical studies and one experimental investigation explored the association between oral microbiota and glioma. Gao et al. [23] reported that glioma patients exhibited significantly worse periodontal parameters compared with both benign tumor patients and population controls. Wang et al. [31] demonstrated that oral microbiota composition differed according to glioma grade, with distinct microbial profiles observed in high-grade versus low-grade tumors. An experimental study further showed that *P. gingivalis* virulence factors, particularly gingipain antigens, were enriched in glioblastoma tissue compared with normal brain samples. Infection of glioma cells induced IL-6 secretion and upregulated PD-L1 expression, suggesting enhanced immune evasion and tumor progression (Figure 3).

Together, these findings support a biologically plausible link between oral pathogens, tumor immune modulation, and glioma progression.

### 3.6. Extracellular Vesicles as Mediators of the Oral–Brain Axis

Two experimental/review studies highlighted the role of extracellular vesicles (EVs) in periodontitis and systemic inflammation. Periodontal pathogens and host cells release EVs containing inflammatory mediators and microRNAs that can influence immune signaling, angiogenesis, and tissue remodeling [32].

**Figure 4:**
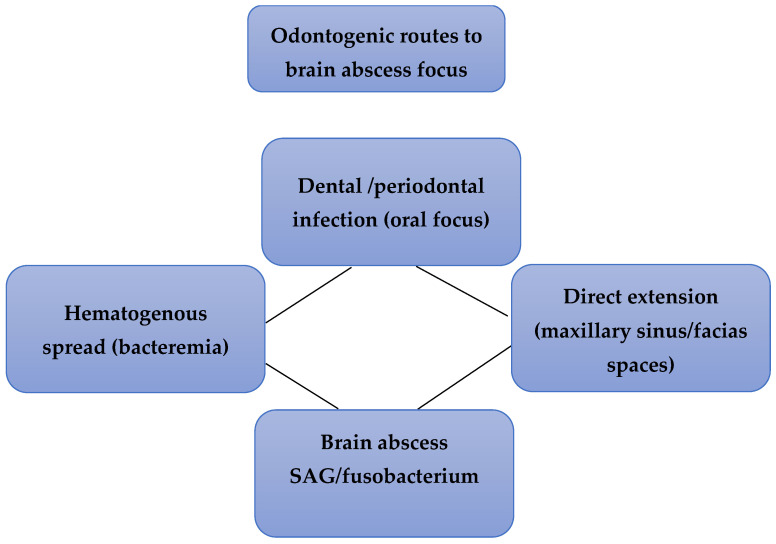
Odontogenic routes leading to brain abscess formation. Oral infections may reach the central nervous system through hematogenous dissemination or direct extension via maxillary sinuses and fascial spaces.

Zhou et al. [20] demonstrated that EVs derived from periodontitis-compromised dental pulp stem cells promoted angiogenic signaling through miRNA-mediated activation of the Hedgehog/Gli1 pathway [24]. These findings suggest that EVs may represent an additional mechanism linking oral inflammation with brain pathology (Figure 4).

### 3.7. Risk of Bias Assessment

**Figure 5:**
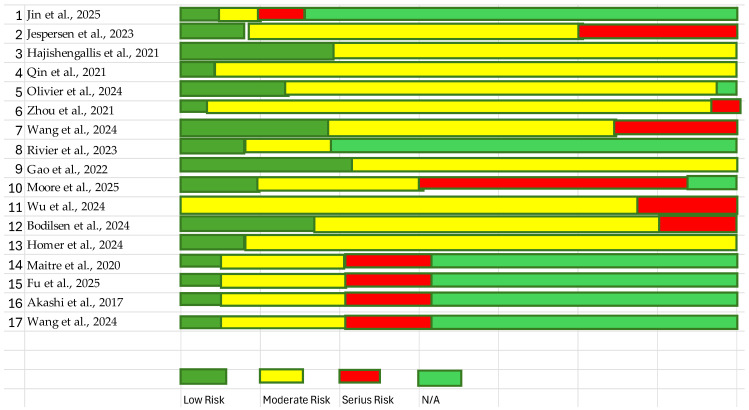
Risk of bias [8,9,10,12,19,20,21,22,23,24,25,26,27,28,29,30,31].

Risk of bias for observational studies was assessed using the ROBINS-I tool. Most observational studies were classified as having moderate risk of bias, primarily due to potential confounding factors, participant selection issues, and heterogeneity in exposure assessment [33]. Genetic and experimental studies were not evaluated using ROBINS-I because this tool is designed specifically for non-randomized clinical research. Their methodological quality was therefore interpreted qualitatively within their respective evidence domains. The overall risk-of-bias evaluation is summarized in Figure 5.

## 4. Discussion

This systematic review synthesized clinical, neuroimaging, genetic, and experimental evidence exploring potential associations between oral diseases and brain pathologies. Across the included studies, three principal evidence domains emerged: (1) clinical evidence linking odontogenic infections with brain abscesses, (2) neuroimaging and genetic studies suggesting associations between oral health and structural brain alterations, and (3) experimental investigations examining biological mechanisms that may connect oral pathogens with neuroinflammatory or tumor-related processes [25]. Taken together, these heterogeneous lines of evidence suggest a potential relationship between oral health and neurological outcomes. However, the strength of evidence varies substantially across domains, and causal inference remains limited [26].

### 4.1. Clinical Evidence Linking Oral Infections and Brain Abscesses

The most consistent evidence identified in this review relates to the association between odontogenic infections and brain abscess formation. Population-based cohorts and retrospective clinical series reported that a substantial proportion of brain abscesses may originate from dental or periodontal sources, particularly in cases classified as cryptogenic. Importantly, several studies emphasized that odontogenic foci may be clinically silent, with chronic dental infections or periodontitis serving as potential reservoirs for intermittent bacteremia [34]. Microbiological investigations frequently identified oral pathogens—particularly members of the *Streptococcus anginosus* group and anaerobic species—in abscess material. Two principal pathways have been proposed to explain this association. The first involves hematogenous dissemination, whereby bacteria originating from periodontal tissues or dental infections enter systemic circulation and subsequently reach the central nervous system. The second pathway involves direct extension, particularly through maxillary sinuses or contiguous anatomical structures [35]. Although these findings support a plausible infectious link between oral disease and cerebral abscess formation, most available evidence derives from observational or retrospective studies. Consequently, residual confounding and incomplete identification of infectious sources remain potential limitations [27].

### 4.2. Oral Health and Structural Brain Alterations

A smaller number of studies examined associations between oral health indicators and structural brain alterations using neuroimaging or genetic approaches [36]. Mendelian randomization analyses suggested that genetically predicted dental caries burden may be associated with reduced cortical thickness in specific brain regions. Similarly, large neuroimaging cohorts reported associations between poor oral health proxies—such as dentures or tooth loss—and markers of white matter injury, including white matter hyperintensities and diffusion abnormalities [37]. These neuroimaging markers are widely interpreted as indicators of cerebral small vessel disease or chronic inflammatory injury. One potential explanation is that persistent oral inflammation may contribute to systemic inflammatory responses, endothelial dysfunction, or vascular alterations that influence brain microstructure. However, these findings should be interpreted cautiously [28]. Oral health indicators in several studies were indirect measures, and important confounding variables—including socioeconomic status, smoking, systemic disease, and healthcare access—may influence both oral health and neurological outcomes [38].

### 4.3. Oral Microbiota and Glioma Biology

Emerging evidence suggests that oral microbiota and periodontal pathogens may influence tumor biology through immunoinflammatory mechanisms. Clinical studies reported differences in periodontal status and oral microbiota composition among glioma patients compared with controls [39]. In addition, experimental studies demonstrated that virulence factors produced by *Porphyromonas gingivalis* can activate inflammatory signaling pathways in glioma cells [40]. In particular, gingipain-associated signaling has been shown to increase IL-6 secretion and PD-L1 expression, mechanisms that may facilitate tumor immune evasion and progression within the tumor microenvironment. Although these findings provide biological plausibility for potential interactions between oral pathogens and glioma biology, the available evidence remains largely experimental. Further clinical and translational studies are required to determine whether these mechanisms operate in human disease [41].

### 4.4. Extracellular Vesicles as Potential Systemic Mediators

Recent experimental research has highlighted extracellular vesicles (EVs) as potential mediators linking localized periodontal inflammation with systemic biological effects. EVs released from periodontal tissues and oral pathogens may contain inflammatory mediators, bacterial components, and microRNAs capable of modulating immune signaling pathways [29]. These vesicles can enter systemic circulation and potentially influence distant tissues. Experimental studies suggest that EV-derived microRNAs may regulate angiogenesis, immune responses, and tissue remodeling through pathways such as Hedgehog/Gli signaling [42]. Although direct evidence linking periodontal EVs with neurological pathology remains limited, these mechanisms provide a biologically plausible framework for systemic communication between oral inflammation and the central nervous system. Future research investigating whether periodontal-derived EVs can influence blood–brain barrier function or neuroinflammatory signaling would help clarify the relevance of this proposed pathway [43].

### 4.5. Bidirectional Oral–Brain Interactions

An important consideration emerging from the literature is that the relationship between oral diseases and brain pathology may be bidirectional rather than exclusively unidirectional [44]. While odontogenic infections may contribute to neurological complications such as brain abscesses, neurological diseases may also influence oral health status. Patients with neurological disorders may experience impaired self-care capacity, reduced manual dexterity, altered immune responses, or systemic illness that predisposes them to worsening oral hygiene and increased susceptibility to dental infections [30]. Consequently, the association between oral and neurological conditions should be interpreted within a broader framework of shared risk factors and reciprocal biological interactions [31].

### 4.6. Strengths and Limitations

This review has several strengths. It integrates multiple evidence domains—including clinical epidemiology, neuroimaging, genetic analyses, and experimental research—providing a comprehensive overview of the proposed oral–brain relationship [45]. The use of a structured narrative synthesis framework also allowed heterogeneous evidence to be interpreted systematically [46]. Nevertheless, several limitations should be acknowledged. The included studies differed substantially in design, exposure definitions, and outcome measures, which prevented quantitative meta-analysis. Many clinical studies were observational, limiting causal inference [47]. Oral health indicators were frequently indirect, and potential confounding factors were not consistently controlled. Additionally, some mechanistic evidence was derived from experimental models or preprint studies that require further validation [48].

### 4.7. Implications for Clinical Practice and Future Research

Despite these limitations, the available evidence highlights the potential relevance of oral health in neurological disease contexts [49,50]. Routine oral evaluation may be particularly important in patients presenting with cryptogenic brain abscesses [51] or in individuals with systemic conditions that increase susceptibility to infection.

Future research should prioritize:

- Longitudinal cohort studies examining oral health and neurological outcomes
- Standardized definitions of oral disease exposure
- Microbiome-based investigations of oral–brain interactions
- Experimental studies exploring systemic signaling pathways linking periodontal inflammation with neurological pathology

Such approaches may help clarify whether oral disease represents a modifiable risk factor influencing neurological health.

Although Mendelian randomization provides genetic evidence reducing confounding bias, it does not replace randomized clinical trials.

## 5. Conclusions

The evidence synthesized in this systematic review suggests a potential association between oral diseases—particularly periodontitis, dental caries, odontogenic infections, and oral microbiota dysbiosis—and several brain pathologies, including brain abscesses, structural brain alterations, and possibly glioma-related biological processes. According to the studies included, clinical investigations indicate that odontogenic infections may represent an underrecognized source of brain abscess formation. Neuroimaging and genetic studies suggest possible links between poor oral health indicators and markers of structural brain injury. Experimental research also provides mechanistic hypotheses involving microbial dissemination, systemic inflammation, immune modulation, and extracellular vesicle signaling. However, the available evidence remains heterogeneous and largely observational, and definitive causal relationships cannot yet be established. Differences in study design, oral health measurements, and neurological outcomes limit direct comparison across studies. Nevertheless, these findings highlight oral health as a potentially relevant component of systemic and neurological health. Improved detection and management of oral diseases may have implications for the prevention of certain neurological complications, particularly in high-risk populations. Future well-designed longitudinal studies, standardized oral health assessments, and mechanistic investigations are required to clarify the nature and direction of the proposed oral–brain relationship.

## 6. Limitations

This systematic review has several limitations. First, there is marked methodological heterogeneity among included studies regarding designs, oral exposure definitions, and brain outcomes, which prevented quantitative meta-analysis and limited direct comparison of results. Second, most studies are observational, restricting causal inference. Some studies also used indirect oral health measures (e.g., tooth loss, denture use, self-reports), introducing classification bias.

Residual confounding by smoking, diet, socioeconomic status, healthcare access, and comorbidities may also affect findings. Additionally, some clinical and microbiological studies had small sample sizes, limiting statistical power. Finally, diverse effect metrics (ORs, RRs, beta coefficients, AUCs) and heterogeneous outcomes (gliomas, abscesses, white matter damage, cortical thickness) hindered quantitative synthesis. Future studies with standardized definitions, larger samples, and longitudinal follow-up are required to strengthen the evidence base.

## Figures and Tables

**Table 1:** Characteristics of included studies (*n* = 16).

Study	Study Design	Population/Sample	Exposure	Neurological Outcome	Evidence Domain	Country
Jin et al., 2025 [8]	Mendelian randomization	GWAS summary data	Genetic liability to glioma	Periodontitis risk	Genetic/epidemiologic	NR
Jespersen et al., 2023 [9]	Population-based cohort	Cerebral abscess patients	Odontogenic focus/oral pathology	Cerebral abscess	Clinical infectious	Denmark
Hajishengallis et al., 2021 [10]	Review	NR	Periodontal disease	Systemic inflammatory pathways	Contextual	USA
Qin et al., 2024 [12]	Case–control + microbiome	HC (*n* = 24), LGG (*n* = 12), HGG (*n* = 23)	Oral microbiota composition	Glioma grade	Microbiome clinical	China
Olivier et al., 2024 [19]	Retrospective cohort	26 brain abscess cases	Chronic dental infection	Brain abscess	Clinical infectious	Germany
Zhou et al., 2021 [20]	Experimental	Dental pulp stem cells	EV microRNA cargo	Angiogenic signaling	Experimental mechanistic	China
Wang et al., 2024 [21]	Mendelian randomization	GLIDE + ENIGMA GWAS	Dental caries (DMFS index)	Cortical thickness	Neuroimaging/genetic	Multi-cohort
Rivier et al., 2024 [22]	Cross-sectional + MR	UK Biobank (*n* = 40,175)	Poor oral health proxy	White matter hyperintensities	Neuroimaging cohort	UK
Gao et al., 2022 [23]	Clinical observation al	Glioma (*n* = 21), benign tumors (*n* = 27), controls	Periodontal parameters (AL, PD)	Glioma presence/grade	Clinical observational	China
Moore et al., 2025 [24]	Experimental + IHC	Glioblastoma tissue models	*P. gingivalis* infection	Glioma immune signaling	Experimental mechanistic	NR
Wu et al., 2024 [25]	Narrative review	NR	Odontogenic infection	Brain abscess	Contextual clinical	NR
Bodilsen et al., 2024 [26]	Nationwide cohort	287 brain abscess cases	Dental infection/oral pathogens	Brain abscess	Clinical infectious	Denmark
Homer et al., 2024 [27]	Retrospective cohort	26 odontogenic abscess cases	Isolated dental focus	Brain abscess	Clinical infectious	Germany
Maitre et al., 2020 [28]	Systematic review	252 studies	Oral microbiota alterations	Brain diseases	Contextual	NR
Fu et al., 2025 [29]	Review	NR	Periodontal extracellular vesicles	Systemic inflammatory signaling	Mechanistic	NR
Akashi et al., 2017 [30]	Case series	3 cases	Silent odontogenic foci	Brain abscess	Clinical infectious	Japan
Wang et al., 2024 [31]	Review	NR	*P. gingivalis* virulence factors	Immune modulation	Mechanistic	NR

Abbreviations: AL = attachment loss; PD = probing depth; HC = healthy controls; LGG = low-grade glioma; HGG = high-grade glioma;

**Table 2:** Main findings and direction of evidence (*n* = 16).

	Key Finding	Direction of Evidence	Evidence Type
Jin et al., 2025 [8]	Genetic liability to glioma associated with increased periodontitis risk	Brain → oral	Mendelian randomization
Jespersen et al., 2023 [9]	Odontogenic foci detected in a significant proportion of cerebral abscess patients	Oral → brain abscess	Population-based cohort
Qin et al., 2024 [12]	Oral microbiota composition differed according to glioma grade	Oral microbiome ↔ brain	Case–control microbiome
Olivier et al., 2024 [19]	Chronic dental infections associated with cryptogenic brain abscess cases	Oral → brain abscess	Retrospective cohort
Zhou et al., 2021 [20]	Periodontitis-derived EVs promote angiogenic signaling	Mechanistic	Experimental
Wang et al., 2024 [21]	*P. gingivalis* virulence factors linked to immune modulation	Mechanistic	Review
Rivier et al., 2024 [22]	Poor oral health linked to white matter abnormalities	Oral → brain structure	Neuroimaging cohort
Gao et al., 2022 [23]	Glioma patients showed worse periodontal parameters than controls	Oral ↔ brain	Clinical observational
Moore et al., 2025 [24]	Gingipain antigens identified in glioma tissue activating IL-6/PD-L1 pathway	Oral pathogen → brain tumor biology	Experimental
Wu et al., 2024 [25]	Odontogenic infections described as possible source of cerebral abscess	Oral → brain abscess	Clinical review
Bodilsen et al., 2024 [26]	Dental infections frequently identified among brain abscess cases	Oral → brain abscess	Nationwide cohort
Homer et al., 2024 [27]	Majority of cryptogenic abscesses showed odontogenic origin	Oral → brain abscess	Retrospective cohort
Maitre et al., 2020 [28]	Evidence linking oral microbiota with neurological diseases	Contextual	Systematic review
Fu et al., 2025 [29]	Extracellular vesicles mediate systemic inflammatory signaling	Mechanistic	Review
Akashi et al., 2017 [30]	Oral pathogens detected in brain abscess despite absence of acute dental symptoms	Oral → brain abscess	Case series
Wang et al., 2024 [31]	Dental caries genetically associated with reduced cortical thickness	Oral → brain structure	Mendelian randomization

**Table 3:** Proposed biological pathways connecting oral diseases with brain pathology.

Mechanistic Pathway	Key Mediators	Evidence Source	Potential Neurological Relevance
Odontogenic infection dissemination	Streptococcus anginous group, *Fusobacterium* spp.	Cohort studies, microbiological analyses	Brain abscess formation
Chronic dental foci and bacteremia	Periodontal inflammation, transient bacteremia	Clinical cohorts, case series	Cryptogenic cerebral abscess
Systemic inflammatory signaling	Cytokines (IL-6, TNF-α), endothelial activation	Observational studies	White matter injury and neuroinflammation
Oral microbiota dysbiosis	Microbial community alterations	Microbiome studies	Glioma severity and tumor microenvironment
*Porphyromonas gingivalis* virulence factors	Gingipains, IL-6, PD-L1 signaling	Experimental models	Tumor immune evasion
Extracellular vesicle signaling	EV-associated miRNA and inflammatory mediators	Experimental studies	Systemic communication and neuroinflammatory pathways
EV-mediated angiogenic signaling	miR-378a, Hedgehog/Gli pathway	Cellular studies	Tissue remodeling and tumor progression

**Table 4:** Incorporated clinical evidence (odontogenic brain abscess and glioma).

Study (Year)	Setting/Design	Sample	Oral Exposure/Focus Definition	Brain Outcome(s)	Key Analytic Findings (Direction + Magnitude)	Notes for Synthesis
Jespersen et al. (2023) [9]	Denmark; population-based cohort (retrospective + prospective data)	44 cerebral abscess patients	“Likely odontogenic” required: oral pathology only infection + oral microbes in pus + radiographic/clinical oral pathology	Cerebral abscess characterization	25/44 (57%) characterized as likely odontogenic; T2D overrepresented (*p* = 0.014) and SAG overrepresented (*p* < 0.01) in odontogenic group	Supports: SAG in brain pus should trigger oral/sinus focus search; adds epidemiologic weight. Cerebral abscesses with odontogenic group
Olivier et al. (2024) [19]	Germany; retrospective single-center (2000–2021)	217 brain abscess (BA) screened; 26 included	Inclusion required no other focus than odontogenic + microbiology consistent with oral origin	BA (clinical course, pathogens, management)	Odontogenic foci diagnosed in 18/26 (69%); SAG pathogens in 21/26 (81%); all surgically treated; 72% had complete/partial neurologic improvement; 3 deaths	Highlights “silent/chronic” oral infections as sufficient trigger; strong clinical signal in cryptogenic BA. Undetected permanent dental infection
Moore et al., 2025 [24]	Experimental + translational (IHC arrays + cell models)	GBM microarrays; U251 glioma + astrocytes	*P. gingivalis* infection; gingipain antigens in GBM tissue	Glioma immune phenotype	Infection induced strong IL-6 response; in U251 cells PD-L1 increased ~30% ± 14% (*p* = 0.0361; *n* = 3); supports IL-6/PD-L1 axis and immune evasion	High mechanistic value; mark as preprint in tables/limitations. Drives *P. gingivalis* infection
Wu et al. (2024) [25]	Review (Acta Neurol Belg)	—	Synthesis of clinical presentation/diagnostics/therapy	Odontogenic BA	Notes insidious onset, diagnostic reliance on microbiology; estimates ~13% BA attributed to odontogenic foci; frequent pathogens include S. intermedius, *F. nucleatum*, *S. anginosus*; main routes: direct extension and hematogenous spread	Use to frame “diagnostic algorithm” and support prevention/early detection. Exploring odontogenic brain abscess Exploring odontogenic brain abscess
Akashi et al. (2016/2017) [30]	Japan; three case reports + literature review	Three cases	Suspected odontogenic foci with no other source (endocarditis/sinusitis excluded)	Brain abscess	Cultures identified oral pathogens including *F. nucleatum*, *Parvimonas micra*, *P. gingivalis*; suspected teeth lacked acute symptoms	Reinforces “occult dental focus” concept; useful for clinical recommendation subsection. Potential brain abscess

## Data Availability

The data presented in this study are openly available in Zenodo 10.5281/zenodo.18773444.

## Supplementary Materials

The following supporting information can be downloaded at https://www.mdpi.com/article/10.3390/biomedicines14040768/s1, Table S1: PRISMA 2020.

## Abbreviations

MRI	magnetic resonance
UK	United Kingdom
EVs	extracellular vesicles
RNAs	ribonucleic acid
